# Effectiveness of Non-Pharmacological Interventions on Stereotyped and Repetitive Behaviors of Pre-school Children With Autism: A Systematic Review

**DOI:** 10.18869/nirp.bcn.8.2.95

**Published:** 2017

**Authors:** Hadi Zarafshan, Maryam Salmanian, Soudeh Aghamohammadi, Mohammad Reza Mohammadi, Seyed-Ali Mostafavi

**Affiliations:** 1. Psychiatry & Psychology Research Center, Tehran University of Medical Sciences, Tehran, Iran.; 2. Department of Psychology, Faculty of Education and Psychology, Shahid Beheshti University, Tehran, Iran.

**Keywords:** Stereotyped behavior, Autistic disorder, Child development disorders, Pervasive, Child, Preschool

## Abstract

**Introduction:**

The present study aimed to review the literature on non-pharmacological interventions used to treat stereotyped and repetitive behaviors by a systematic method.

**Methods:**

Two authors independently performed a search strategy on Medline/PubMed, Scopus and PsycINFO on English articles published up to April 23, 2014 with relevant search keywords. We also reviewed the bibliographies of retrieved articles and conference proceedings to obtain additional citations and references. We examined those articles that addressed non-pharmacological interventions on reducing stereotyped and repetitive behaviors in preschool children with autism. Four independent reviewers screened relevant articles for inclusion criteria and assessed the quality of eligible articles with CONSORT checklist.

**Results:**

In our search, 664 relevant articles were found. After removing duplicates and screening based on title, abstract, and full text, 15 high-quality studies were finally included in data analyses. The included articles were published from 1987 to 2013. Three studies were designed as A-B, two as A-B-A, and reminders as A-B-A-B. The data and results of 3 clinical trials were synthesized; two of them were parallel randomized clinical trial and another one was designed as cross-over. Interventions were completely heterogeneous in case studies, including non-contingent auditory stimulation, response interruption and redirection, teaching the children to request assistance on difficult tasks, family-implemented treatment for behavioral inflexibility with treatment approach, vocal or motor response interruption and redirection, brushing, water mist treatment, exposure response prevention, tangible reinforcement or social reinforcement, and music. Interventions in clinical trials included touch therapy, kata techniques training program, and aerobic exercise.

**Conclusion:**

The results of our review indicate that different kinds of non-pharmacological interventions can be used to treat repetitive behaviors in children with autism; however, sufficient evidence for their effectiveness does not exist. Future research using more precise methods (RCTs) can clarify which methods and techniques are effective in reducing repetitive behavior of children with autism.

## 1. Introduction

Autism Spectrum Disorders (ASDs) are neurodevelopmental disorders with poorer cognitive performance that affect about 1% of population and characterized by impairments in three domains of function; social interaction, communication, and Repetitive or Stereotyped Behaviors (RSBs) (American Psychiatric Association, 2013; Lai, Lombardo, & Baron-Cohen, 2014; Mohammadi, Salmanian, & Akhondzadeh, 2011; Tehrani-Doost, Salmanian, Ghanbari-Motlagh, & Shahrivar, 2012; Salmanian, Tehrani-Doost, & Shahrivar, 2012). Repetitive or Stereotyped Behaviors (RSBs) refer to vocal or motor behaviors (Matson, Kiely, & Bamburg, 1997; Smith, & Van Houten, 1996) and manipulation of objects (Falcomata, Roan Feeney, & Stephenson, 2010) without any apparent function (Singer, 2009). These behaviors have negative effects on learning and social capacity of the people with ASDs (Pierce & Courchesne, 2001; Nadig, Lee, Singh, Bosshart, & Ozonoff, 2010) and also negatively affect function, well-being, stress level, and parenting of their families (Bishop, Richler, Cain, & Lord, 2007; Lounds, Seltzer, Greenberg, & Shattuck, 2007; Shattuck et al., 2006; Greenberg, Seltzer, Hong, & Orsmond, 2006).

Repetitive and stereotyped behaviors (RSBs) are common in people with autism (around 90%) and begin around 3–4 years of age (Hollander et al., 2004; Watt, Wetherby, Barber, & Morgan, 2008) and continue through the life. Given the significant negative effects of RSBs on people with autism and its early emergence, finding relevant interventional methods to treat and reduce RSBs are crucial.

Generally, interventions in the field of autism are divided into two broad categories; comprehensive models and focused practices (Odom, Boyd, Hall, & Hume, 2009). Comprehensive models address different developmental and behavioral skills in children with autism, while, focused practices address specific skills or symptoms (Boyd, McDonough, & Bodfish, 2011).

Many different therapeutic strategies are used to manage RSBs. Pharmacotherapy is the most studied method that is shown to be effective on the reduction of symptoms among children with autism in clinical trial studies (Pearson et al., 2013; Mohammadi et al., 2013; Klaiman, Huffman, Masaki, & Elliott, 2013). However, evidence shows a high risk of negative effects, including weight gain, sedation, and extrapyramidal effects (Weitlauf et al., 2001). Side-effects of pharmacotherapy are the main reason which calls for developing non-pharmacological interventions such as self-management (Mancina, Tankersley, Kamps, Kravits, & Parrett, 2000), cognitive-behavioral therapy (Gajdzik, & Brynska, 2012), behavioral therapy (Conroy, Asmus, Sellers, & Ladwig, 2005; Britton, Carr, Landaburu, & Romick, 2002), and parent training (Scahill et al., 2012; Pajareya & Nopmaneejumruslers, 2011). In the meantime, there is no census over the relevant and usage of these methods. The present study explores the literature on nonmedical interventions that are used to treat RSBs by systematic method to better understand this field.

## 2. Methods

### 2.1. Databases and search strategy

Two authors independently performed a search strategy on Medline/PubMed, Scopus, and PsycINFO for English-language studies published up to 23 April, 2014 with following search terms; (“Autistic Disorder” OR “Autism spectrum disorder” OR “Pervasive developmental disorder” OR “Asperger Syndrome”) AND (“Stereotyped Behavior” OR “Motor Stereotypies” OR “Repetitive Behavior” OR “Repetitive use of object” OR “Repetitive speech”) AND (Treatment OR Intervention OR Therapy). We customized our search term for every database and used specific terms for “stereotyped and repetitive behaviors” based on DSM-5 description of ASDs. We also reviewed the bibliographies of retrieved articles and conference proceedings to obtain additional citations and references.

### 2.2. Study selection criteria

English-language case studies and clinical trials were considered eligible for this analysis if they dealt with ASD in preschool age period (under 7 years). We used those articles that addressed any non-pharmacological interventions on reducing stereotyped and repetitive behaviors in preschool children with autism. Furthermore, the qualified studies had to report sufficient statistics for data syntheses. Exclusion criteria comprised all articles that dealt with pharmacological, traditional, dietary, and herbal interventions.

### 2.3. Study selection and data extraction methods

Four independent reviewers screened relevant articles for inclusion criteria and assessed the quality of eligible articles with CONSORT checklist. High quality studies were included in data syntheses. Authors extracted data of each article onto extraction forms prepared in Microsoft Excel 2007. Variables which were extracted from case studies included study design, intervention, outcome, cases age (month)/sex, intervention sessions, mean baseline, and outcome values. Variables which were abstracted from clinical studies included study design, number of participants in each intervention and control groups, mean age of participants in each group, intervention duration, outcome, and results.

### 2.4. Data analyses

The included studies were homogeneous with respect to design and participant characteristics. The data, results, and conclusions of case studies were synthesized separately from data and results of clinical trials. In the case of articles in which multiple interventions had been performed (e.g. ERIN N. AHRENS et al. 2011) experiments were extracted separately. Because of lack of clinical trials on this field and insufficient data, we were not able to perform further statistical and meta-analyses.

## 3. Results

**Figure 1.**
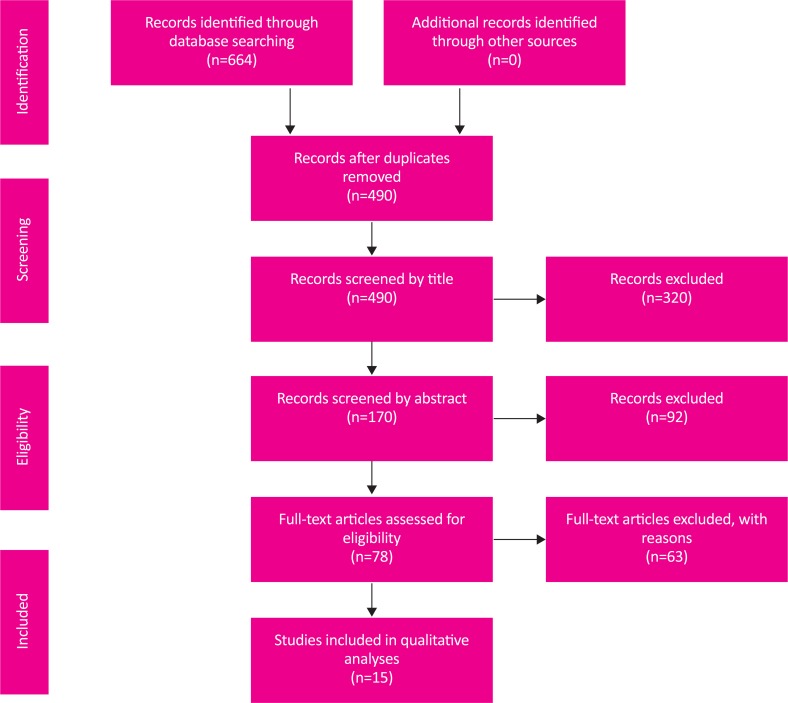
Flowchart for selection procedure of articles.

Six hundred and sixty-four articles were found and inserted into End Note software ver. X6. After removing duplicate articles retrieved by two search engine databases, 490 articles were remained in the study (Figure 1). Then, authors reviewed the remained articles by titles and then by abstracts to check for potentially relevant studies. Afterward, 4 authors independently, assessed the quality of 78 eligible full texts with CONSORT checklist. Finally, 15 high-quality studies were included in data analyses.

The included articles were published from 1987 to 2013. The data and results and conclusions of 12 case studies were presented in Table 1 (ERIN N. AHRENS et al. 2011 has allocated 2 lines in tables due to multiple experiments and outcomes). Three studies designed as AB, two as ABA, and reminders as ABAB. In these case studies, baseline and final points each included several sections, in which investigators observed and counted the number of stereotyped behavior. In Table 1, mean baseline refers to mean number of stereotyped behavior for all sections at baseline. Similarly, the mean outcome refers to mean number of stereotyped behavior for all sections at final point. The data and results of 3 clinical trials were presented separately in Table 2. Two of them were randomized clinical trial and another one was designed as cross-over clinical trial.

**Table 1.** Case reports of non-pharmacological interventions on stereotyped and repetitive behaviors in preschool children with autism.

**Author, Year**	**Study Design**	**Quality of Article %**	**Intervention**	**Outcome**	**Cases Age (Mon/Sex)**	**Intervention Sessions**	**Mean Baseline 1**	**Mean Outcome 1**	**Mean Baseline 2**	**Mean Outcome 2**	**Results**
Saylor et al. (2012)	AB	65	Non-contingent auditory stimulation	Vocal stereotypy	79	30	55	42			Inconsiderable improvement
					66	30	71	65			
Schumacher et al. (2011)	AB	65	Response interruption and redirection	Vocal stereotypy	60/F	30	5	3			Inconsiderable improvement
Durand et al. (1987)	AB	60	Teaching the children to request assistance on the difficult tasks	Motor stereotypy	84/M	25	24.70	3.80			Considerable improvement
Ahearn et al. (2007)	ABAB	95	Response interruption and redirection (RIRD)	Vocal stereotypy	36/M	20	45.60	11.20	44.60	8.30	Considerable improvement
					84/F	16	91	33.30	87	20.85	
					84/F	14	48	12.60	23.30	4.30	
Ahrens et al. (2011)	ABAB	100	Vocal response interruption and redirection (RIRD)	Vocal stereotypy	72/M	40	59	31	-	-	Considerable improvement
					48/M	80	30	9.8	-	-	
					60/M	50	81	62	87	49	
					48/M	50	82	28	88	34	
Ahrens et al. (2011)	ABAB	100	Motor response interruption and redirection (RIRD)	Vocal stereotypy	72/M	40	59	35	-	-	Considerable improvement
					48/M	80	30	23	-	-	
					60/M	50	81	42	87	39	
					48/M	50	82	18	88	27	
Boyd et al. (2011)	ABAB	90	Family-implemented treatment for behavioral inflexibility (FITBI) with treatment approach: Response interruption and redirection (RIR)	Repetitive behaviors	40/M	12	98	27	97	34	Considerable improvement
					39/M	12	66	29	63	26	
					44/M	12	68	23	59	10	
					65/M	12	69	39	76	45	
					53/M	12	62	21	75	24	
Cassella et al. (2011)	ABAB	90	Response interruption and redirection (RIRD)	Vocal stereotypy	59/M	35	73.85	30.62	64.6	37	Considerable improvement
Davis et al. (2011)	ABA	75	Brushing protocol: phase 1: Week 3 of brushing, phase 2: Week 5 of brush in	Stereotyped behavior	48/M	20	40	52			Worsening effect
Bailey et al. (1983)	ABA	85	Water mist treatment	Stereotypic behavior: mouthing behavior	84/M	91	43.5	5.1			Considerable improvement
Boyd et al. (2013)	AB	80	Exposure response prevention (ERP)	Repetitive behaviors	60/M	28	14(N)	12(N)			May partially be effective
					72/M	28	6(N)	7(N)			
Kang et al. (2013)		78	Tangible reinforcement Social reinforcement	Stereotyped behavior	36/M	11		94			Considerably higher levels of stereotyped behavior while accessing tangible reinforcers than when accessing social reinforcers
					48/M	11		74			
				Stereotyped behavior	36/M	11		7.5			
					48/M	11		0			
Lanovaz, (2011)	ABAB	80	Music	Vocal Stereotypy	60/F	28	47.8	25.16	74.16	31.12	Considerable improvement
					72/M	28	68.75	33.33	62.83	24.75	

**Table 2.** RCTs of non-pharmacological interventions on stereotyped and repetitive behaviors in preschool children with autism.

**Author, Year**	**Study design**	**Quality of Article %**	**Number of Intervention Group (Mean Age)**	**Number of Control Group (Mean Age)**	**Intervention**	**Intervention Duration**	**Outcome**	**Results**
Field et al. (1997)	RCT	80	11(54)	11(54)	Touch therapy	4 Weeks	Stereotypic behaviors	Significant improvement compared to control group
Bahrami et al. (2012)	RCT	65	15(110)	15(108)	Kata techniques training program	56 Sessions	Stereotypic behaviors	Significant improvement compared to control group
Oriel et al. (2011)	Cross-over clinical trial	75	9(62)	9(62)	Aerobic exercise	3 Weeks	Stereotypic behaviors	No significant improvement

### 3.1. Participants

Thirty-nine participants were included in 12 case studies (stereotypy in 4 of them were measured in two times one after vocal and then after motor response interruption and redirection (RIRD) ERIN N. AHRENS et al. 2011). Four participants were female and others (most of them) were male.

### 3.2. Interventions and outcomes

Interventions were completely heterogeneous in case studies, including non-contingent auditory stimulation, response interruption and redirection, teaching the children to request assistance on difficult tasks, family-implemented treatment for behavioral inflexibility (FITBI) with treatment approach, vocal or motor response interruption and redirection (RIR), brushing, water mist treatment, exposure response prevention (ERP), tangible reinforcement/social reinforcement, and music. Interventions in clinical trials included touch therapy, kata techniques training program, and aerobic exercise. However, the outcomes were vocal (6 studies) or motor stereotypy.

## 4. Discussion

Apparently, many different techniques were used in treating behavioral stereotypies in children with autism. These techniques are based on different approaches; mostly behavioral (Schumacher & Rapp, 2011; Ahearn, Clark, MacDonald, & In Chung, 2007; Ahrens, Lerman, Kodak, Worsdell, & Keegan, 2011; Boyd, McDonough, Rupp, Khan, & Bodfish, 2010; Cassella, Sidener, Sidener, & Progar, 2011; Kang et al., 2013; Boyd, Woodard, & Bodfish, 2013) and sensory (Saylor, Sidener, Reeve, Fetherston, & Progar, 2012; Davis, Durand, & Chan, 2011; Bailey, Pokrzywinski, & Bryant, 1983; Field et al., 1997) approaches. The results show that almost all techniques have positive effects on reduction of behavioral stereotypies, but surprisingly two of them had both positive and negative effects Boyd, Woodard, & Bodfish, 2013; Ahearn et al., 2007).

Behavioral approaches are based on the idea that positive response reinforces a behavior, but negative response suppresses the original behavior (Iwata, Dorsey, Slifer, Bauman, & Richman, 1982; Iwata et al., 1994). In regards to stereotyped behaviors, investigators think that stereotyped behaviors are self-motivated and maintained because of their positive result on children with autism (Asmus, Ringdahl, Sellers, Call, Andelman, & Wacker, 2004). Finding and changing the consequences of stereotyped behaviors can decrease these behaviors (Rapp, 2006; 2007). Included studies in our review have reported similar findings. In these studies, researchers that manipulated the consequences of behaviors could change undesirable behaviors (Schumacher, & Rapp, 2011; Ahrens et al., 2011).

Another theory in explaining stereotyped behavior claimed that impairment in functional communication would lead to repetitive and stereotyped behaviors (Mandy & Skuse, 2008); children with autism who cannot communicate with environment have several unmet needs that cause inappropriate behaviors (Sigafoos & Meikle, 1996). Based on our review, Durand found that teaching children with autism to request help, reduced motor stereotypy (Durand & Carr, 1987).

Sensory dysfunctions are common in children with autism and can be categorized as hyper-responsiveness, hypo-responsiveness, and sensory seeking (Boyd et al., 2010). The association between sensory dysfunction and repetitive behaviors has been explored in several studies (Gabriels et al., 2008; Boyd, McBee, Holtzclaw, Baranek, & Bodfish, 2009). These studies have shown a significant association between sensory dysfunction and stereotypy that can be considered in interventions (Boyd et al., 2010). Some studies that we found were in line with this idea. They revealed that interventions that have been considered sensory issues would decrease repetitive behaviors (Schumacher, & Rapp, 2011; Bailey, Pokrzywinski, & Bryant, 1983; Field et al., 1997; Lanovaz, Sladeczek, & Rapp, 2011).

As seen above, interventions that have focused on positive reinforcement (tangible reinforcement, social reinforcement), improvement of communication skills (teaching kids to request help) and sensory problems (brushing protocol, water mist treatment, and touch therapy) are most effective. These methods are supported by previous literature in the field of autism. Numerous studies have shown that using principles of applied behavior analysis (ABA) is effective in reduction of autism symptoms. Core principle of ABA is positive reinforcement. Our review shows that using positive reinforcements decreases stereotypic behaviors. Sensory processing problems and communication difficulties are considered as underpinning of stereotypic behaviors in people with autism. Our review also shows that intervention that focused on these area have successfully decreased stereotypic behaviors.

An important issue that should be considered in interpretation of the results of these studies is related to their case study designs which their results do not reveal reliable evidence. Only three of them were RCT and used intervention except behavioral interventions. Intensity of disease, duration of intervention, number of sections and therapist experiments can predict response to nonpharmacological treatment.

Non-pharmacological interventions on stereotyped and repetitive behaviors in preschool children with autism was based on sensory and behavioral techniques. The results show that almost all these techniques have positive effects on reduction of behavioral stereotypies, but surprisingly two of them (tangible and social reinforcement, and brushing protocol) had both positive and negative effects.

### 4.1. Suggestions

Most current studies have used single subject design that leads to inability to control many confounders. Thus, we can’t precisely infer that which method can decrease stereotypic behaviors in autistic children better than other methods. Also, there is no comparison between methods in current studies. Well-designed study protocol and comparison between different methods is necessary to direct clinicians and parents to choose the best method. In addition, specific characteristics of children with autism should be considered. Some of them show stereotypic behaviors due to sensory processing problems, some due to communication problems, and some for behavioral problems. Future studies should carefully evaluate participants and clarify which methods better decrease stereotypic behavior in any subtype. Furthermore, research using more precise methods (RCT) can clarify what methods and techniques are effective in reducing repetitive behavior of children with autism. Also previous studies have revealed that autism phenotypes may predict psychological adjustments in parents and developing siblings (Mohammadi, Zarafshan, & Ghasempour, 2012; Mohammadi & Zarafshan, 2014), so we suggest that future trials on stereotypy behaviors also consider psychological effects on families of children with ASDs.

### 4.2. Limitations

Poor accessibility to full text of articles and insufficient data for performing meta-analysis are the main limitations for this study.
